# Healthcare deprivation matters: a novel framework to unveil the influencing mechanisms of aging anxiety and healthcare utilization

**DOI:** 10.3389/fpubh.2025.1679262

**Published:** 2025-10-14

**Authors:** Zhiyi Luo, Fanyi Kong, Jianhua Wu, Zihao Bian, Yuxuan Li, Jinli Yang, Yansui Yang, Yuehua Liu

**Affiliations:** ^1^School of Healthcare Management, Tsinghua Medicine, Tsinghua University, Beijing, China; ^2^Wireless and Computing Product R&D Institute, ZTE Corporation, Shenzhen, China; ^3^School of Economics, Peking University, Beijing, China; ^4^Medical School, Shenzhen University, Shenzhen, China; ^5^Dong Fureng Institute of Economic and Social Development, Wuhan University, Wuhan, China

**Keywords:** aging anxiety, healthcare utilization, healthcare deprivation, double machine learning, mediation effect

## Abstract

**Objectives:**

Aging anxiety is not only a health issue but also a stress response to the structural risk of insufficient medical resources. This study aims to reveal the impact of aging anxiety on individual healthcare utilization and the complex psychosocial mechanisms behind it.

**Methods:**

Based on large-scale data from the 2021 Chinese General Social Survey (CGSS), the study employs a Double Machine Learning (DML) method to build a causal inference model. The random forest algorithm is used to estimate the marginal effect of aging anxiety on healthcare utilization. The robustness checks and placebo tests are conducted to further verify the model’s stability and validity. Finally, heterogeneity analysis explored the differential impact of independent variables across groups by age, education, household health status and kid number.

**Results:**

Aging anxiety has a significant positive effect on healthcare utilization (*β* = 0.110, *t* = 4.895). It mediates through multiple pathways including healthcare accessibility anxiety (*β* = 0.344, *t* = 16.904), affordability anxiety (*β* = 0.384, *t* = 19.845), physical deterioration (*β* = 0.160, *t* = 7.286), psychological pessimism (*β* = 0.175, *t* = 7.819), sleep disorder (*β* = 0.104, *t* = 6.124), and self-efficacy loss (*β* = 0.160, *t* = 5.595). Heterogeneity analysis shows significant differences in this effect across groups with different socio-demographic characteristics and health statuses, reflecting variations in medical demand and anxiety responses among populations.

**Conclusion:**

To alleviate anxiety related to medical resource shortage and promote healthy aging, a multidimensional response system is needed. This includes improving medical insurance, advancing primary healthcare management, enhancing health literacy, and building family-community support networks. Policy design should emphasize the synergy between psychosocial factors and institutional frameworks, providing theoretical and empirical support for equitable, inclusive healthcare utilization and sustainable health development.

## 1 Introduction

Globally, the average life expectancy has risen dramatically, from 46.5 years in 1950 to 73.2 years in 2024, according to *the World Population Prospects (2024)* (1). Preliminary estimates suggest this will reach 75 years by 2030. By the late 2070s, the number of people aged 65 and older is expected to surpass those under 18, reflecting a profound demographic shift toward population aging. As individuals live longer, societies face new challenges related not only to physical health and economic sustainability, but also to psychological and social well-being in later life.

Within this broader context, aging anxiety, defined as individuals’ fear or worry about aging and its potential negative consequences (2), has emerged as a critical public health issue. While aging itself is a natural biological process, anxiety related to it is deeply influenced by cultural narratives and social environments (3). Traditional cultural views frequently associate old age with decline, loneliness, frailty, and social marginalization, perpetuating negative stereotypes (4) that are internalized across the life course. These pervasive and persistent negative portrayals exacerbate feelings of vulnerability and loss of control (5), feeding into the sociocultural foundations of aging anxiety. In this way, aging anxiety reflects not only personal concerns about health and mortality, but also broader societal attitudes and structural inequalities that shape how aging is experienced and anticipated. Furthermore, aging anxiety is not limited to physiological or emotional concerns; it also fundamentally influences individuals’ health-related decisions and behaviors. As anxiety about aging encompasses fears of health deterioration, loss of autonomy, and economic insecurity (6), it naturally shapes how people perceive their current and future healthcare needs. In addition, aging anxiety is not limited to the older adults. Studies show it is widespread across all age groups (7), and often peaking in middle age (8). Hence, this psychological state thus plays a crucial role in driving healthcare utilization patterns across the lifespan (9).

Healthcare utilization, the frequency and nature of people’s engagement with medical services, serves as a vital indicator of health system responsiveness and population well-being (10). Research finds a negative link between aging anxiety and physical symptoms, with self-perceived health acting as a mediator that influences healthcare and public service utilization (11). Health behaviors (12) and anxiety (9) also play a role in healthcare utilization. Understanding the factors that influence healthcare utilization is essential, as it directly affects disease prevention, early diagnosis (13), treatment adherence, and health outcomes (14). In the context of aging populations, clarifying how aging anxiety impacts healthcare utilization is particularly important. It informs whether anxiety leads to timely, appropriate medical care or whether it results in either overuse or underuse of services, both of which have significant implications for individual health and healthcare system sustainability. Additionally, the concept of healthcare deprivation highlights the multifaceted barriers, both structural and psychological, that modify the relationship between aging anxiety and healthcare utilization (15). Healthcare deprivation can be described as the situation where individuals do not have access to medical services when needed (16). Given the demographic trends worldwide, investigating the pathways from aging anxiety to healthcare utilization is crucial for designing effective, equitable health policies.

Nevertheless, most existing research on aging anxiety and healthcare utilization has been conducted in developed countries such as the United States, South Korea, and Germany (3, 17), with limited focus on developing contexts. Moreover, while studies have explored how depression, general anxiety (18), and death anxiety (19) affect healthcare utilization, aging anxiety remains understudied. These gaps are significant given the scale and speed of demographic aging in countries like China. China, in particular, exemplifies the urgency of this issue (20). In 2024, the population aged 60 and above reached 310 million, nearly equal to the entire population of the United States, and within this group, 220 million are aged 65 or older, constituting 15.6% of the national population. This immense and rapidly growing older adults demographic heightens the significance of aging anxiety as a social and health challenge. China’s demographic transition provides a critical context for studying the psychological mechanisms of aging anxiety, its mediating factors, and the resulting patterns in healthcare utilization.

In all, aging anxiety is not merely a mental health concern, but also a social sustainability issue. It intersects with key targets of the United Nations’ 2030 Sustainable Development Goals (SDGs), especially Goal 3: “Ensure healthy lives and promote well-being for all at all ages.” Therefore, advancing research on aging anxiety and healthcare utilization can provide important evidence to guide the design of fairer and more inclusive policies that support healthy aging and equitable access to healthcare services.

## 2 Materials and methods

### 2.1 Building DML model

We employed the Double Machine Learning (DML) method to estimate the causal effect of aging anxiety on healthcare utilization. This approach was chosen over traditional econometric methods due to its superior ability to handle high-dimensional confounders and reduce model misspecification bias to obtain unbiased causal effect estimation (21). This method builds upon the foundational principles established by Chernozhukov et al. (22), emphasizing its capability to incorporate a data-driven control of observed confounders, which enables valid inference under specific regularity conditions. In our study, the DML model follows a two-step process: first, we estimate control variables (such as age, education, and health status) to isolate the true effect of aging anxiety; second, we use machine learning algorithms to estimate the treatment effect of aging anxiety on healthcare utilization while adjusting for these control variables. The residual error from this treatment step is then treated as an instrumental variable, enabling the calculation of the unbiased causal effect. We constructed the model based on formulas adapted from related literature. A partial linear model was initially formulated as follows (Equations 1–6):


(1) $$\;{\mathrm{HU}}_{i}=\theta_{0}\cdot {\mathrm{AA}}_{i}+g(X_{i})+U_{i}\;E(U_{i}|,{\mathrm{AA}}_{i}|,X_{i})=0$$

Where ${\mathrm{HU}}_{i}$ represents healthcare utilization for individual i. ${\mathrm{AA}}_{i}$ is the aging anxiety indicator, and $X_{i}$ consists of control variables. The function $g(X_{i})$ estimates these controls, while $U_{i}$ is the error term, and $\theta_{0}$ represents the treatment effect coefficient. Next, we estimate the treatment effect and consider potential bias as follows:


(2) $${\hat{\theta }}_{0}={\left(\frac{1}{n}\sum_{i\in I}\;\;{\mathrm{AA}}_{i}^{2}\right)}^{-1}\frac{1}{n}\sum_{i\in I}\;\;{\mathrm{AA}}_{i}({\mathrm{HU}}_{i}-\hat{g}(X_{i}))$$

(3) $$\begin{array}{l} \sqrt{n}({\hat{\theta }}_{0}-\theta_{0})={\left(\frac{1}{n}\sum_{i\in I}{\mathrm{AA}}_{i}^{2}\right)}^{-1}\frac{1}{\sqrt{n}}\sum_{i\in I}{\mathrm{AA}}_{i}U_{i}+{\left(\frac{1}{n}\sum_{i\in I}{\mathrm{AA}}_{i}^{2}\right)}^{-1}\\ \frac{1}{\sqrt{n}}\sum_{i\in I}{\mathrm{AA}}_{i}[g(X_{i})-\hat{g}(X_{i})] \end{array}$$

To accelerate convergence and mitigate biases introduced by regularization, we introduced the following auxiliary regression model:


(4) $$\begin{array}{c} {\mathrm{AA}}_{i}=m(X_{i})+V_{i}\;E(V_{i}|X_{i})=0 \end{array}$$

Where $m(X_{i})$ is the regression function for aging anxiety, $\hat{m}(X_{i})$ use a machine learning algorithm to estimate, and $V_{i}$ is the residual error term. After partialling out the effect of the control variables from aging anxiety and obtaining a preliminary estimate of $\hat{g}(X_{i})$ from the auxiliary sample, we treat ${\hat{V}}_{i}$ it as the instrumental variable for aging anxiety and then calculate the unbiased estimator ${\hat{\theta }}_{0}$ using the main sample of observations:


(5) $$\begin{array}{c} {\hat{\theta }}_{0}={\left(\frac{1}{n}\sum_{i\in I}\;\;{\hat{V}}_{i}\cdot \mathrm{AA}\right)}^{-1}\frac{1}{n}\sum_{i\in I}\;\;{\hat{V}}_{\mathrm{it}}({\mathrm{HU}}_{i}-\hat{g}(X_{i})) \end{array}$$

(6) $$\begin{array}{c} \begin{array}{l} \sqrt{n}({\hat{\theta }}_{0}-\theta_{0})={\left[E(V_{i}^{2})\right]}^{-1}+\frac{1}{\sqrt{n}}\sum_{i\in I}V_{i}\cdot U_{i}+{\left[E(V_{i}^{2})\right]}^{-1}\\ \frac{1}{\sqrt{n}}\sum_{i\in I}[m(X_{i})-\hat{m}(X_{i})][g(X_{i})-\hat{g}(X_{i})] \end{array} \end{array}$$

### 2.2 Data description

The data of this study is from the 2021 Chinese General Social Survey (CGSS), a nationally representative cross-sectional social survey by Renmin University of China (23). It systematically collects detailed information on multiple levels, such as aging, health service, social perception, and individual characteristics, which can analyze whether aging anxiety can influence healthcare utilization. Based on a rigorous multi-stage stratified probability sampling design, the CGSS 2021 has a total of 8,148 samples, covering 19 provinces, municipalities, and autonomous regions in China (20, 24). We primarily use data from the core module, thematic module, and the EASS health module ac-cording to our research’s theme and purpose. Samples with missing data on key variables are excluded, so 2,663 participants from the CGSS 2021 are available for the following analysis. Because we utilize de-identified public data from secondary dataset with ethical approval, no additional ethics review is required.

### 2.3 Variable measurement

The primary dependent variable is healthcare utilization. It refers to the frequency with which individuals access formal medical services for diagnosis, treatment, or preventive care. Therefore, we operationalized this concept using respondents’ self-reported answer to the question “How often did you seek medical care for your own health in the past year?.” Based on the survey responses, we constructed a six-category ordinal variable where higher values indicate greater healthcare utilization frequency.

The independent variable in this study is aging anxiety. Since comprehensive instruments like the Aging Anxiety Scale (AAS) (2) or the Personal Anxiety Toward Aging Scale (PAAS) (25) are absent in large-scale Chinese surveys, we adopted a validated alternative approach using three questions derived from the health module in the CGSS 2021. These items capture core concerns about physical disability/mobility, loss of cognitive ability or autonomy in decision-making, and financial dependence—key dimensions identified in established aging anxiety scale. Following methodological precedents, we constructed a continuous variable through averaging the sum of these items’ responses, where higher scores indicate more pronounced aging anxiety. The specific questions are in Appendix Table S1. We also conducted a Pearson correlation test to assess the relationship between the dimensions of the aging anxiety variable and itself, with results indicating that both are significantly correlated and directionally consistent (Appendix Table S2).

To validate the measurement of aging anxiety, we performed a KMO test and Bartlett’s Test of Sphericity, which confirmed the data’s suitability for factor analysis (Appendix Table S3). The KMO value was 0.693, indicating an adequate sample size, and Bartlett’s Test yielded a significant result (chi-square = 2034.836, *p* < 0.01), further supporting the appropriateness of the data for factor analysis. Principal Component Analysis (PCA) revealed that the first extracted factor accounted for 68.058% of the variance (Appendix Table S4). The factor loadings for the subscales of aging anxiety were as follows: 0.821 for physical frailty aging anxiety, 0.845 for cognitive decline aging anxiety, and 0.809 for financial dependency aging anxiety. These results confirm the construct validity of the aging anxiety scale (Appendix Table S5).

**Figure 1:**
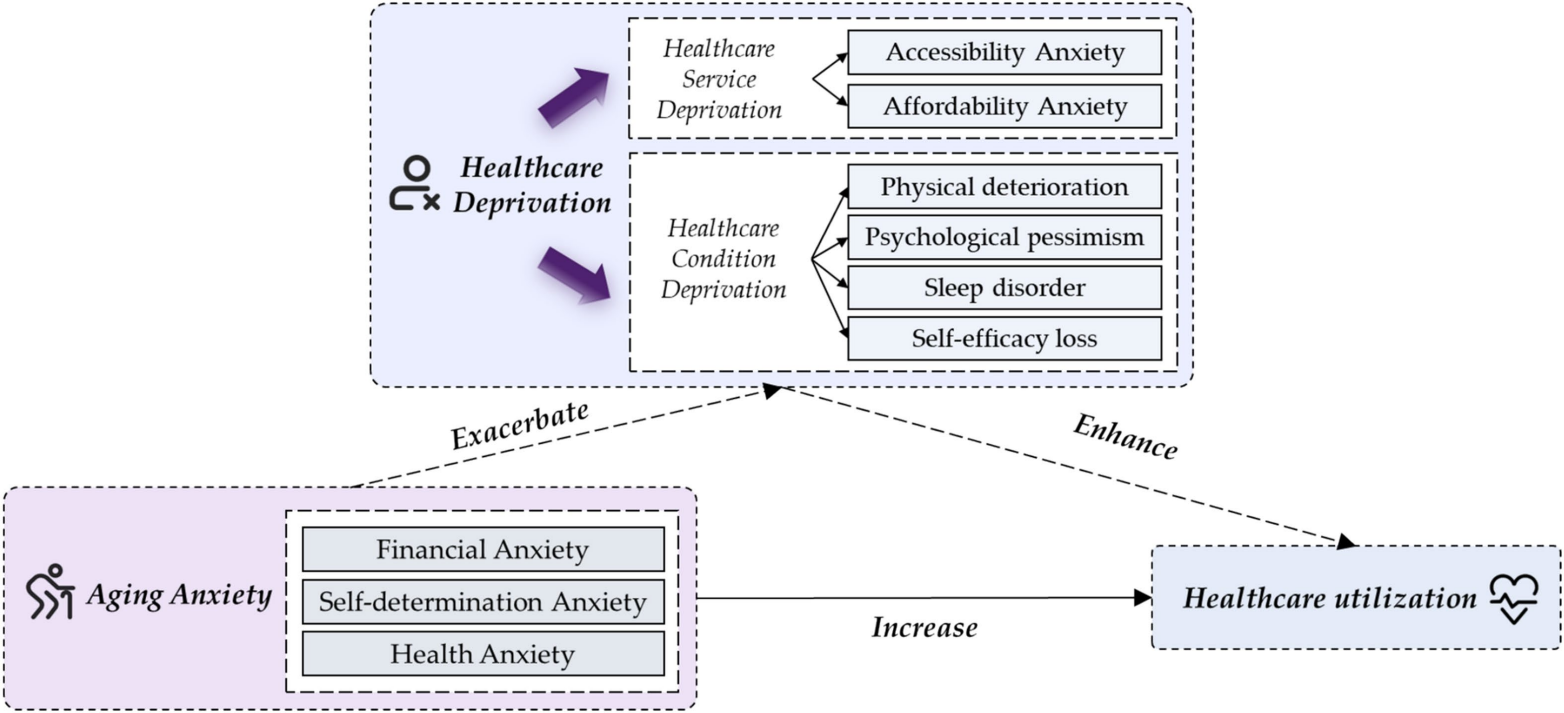
The influencing framework.

The influencing framework of aging anxiety on healthcare utilization is theoretically anchored in health demand theory (26) and perceived health theory (27), with extensions to clarify the dual pathways linking aging-specific psychological factors to healthcare behaviors (Figure 1). Guided by health demand theory, which posits that perceived access barriers and cost concerns shape healthcare-seeking motives, we proposed the healthcare service deprivation pathway. Aging anxiety heightens worries about health service accessibility and economic burdens, driving preventive healthcare utilization as a preemptive response to potential service inadequacies. Drawing on perceived health theory, which emphasizes the role of subjective health perception in triggering health actions, we developed the healthcare perception deprivation pathway. Aging anxiety impairs self-perceived physical / psychological wellbeing, sleep quality, and healthcare self-efficacy, thereby prompting diagnostic and therapeutic healthcare-seeking to address perceived health deteriorations. In essence, this dual-pathway model highlights that operationalizing healthcare deprivation as a core mediating mechanism offers a novel and critical perspective to unravel the intricate causal link between aging anxiety and healthcare utilization. The detailed measurements of mediating variables could be found in Appendix Table S1.

Other socio-demographic control variables were included to avoid potential effects from confounding factors, including age, gender, religion, educational status, marital status, political status, kid number, housing number, and living environment. The specific definitions and measurements can also be found in Appendix Table S1.

### 2.4 Statistical analysis

All of data analyses were conducted using the Double ML package (version 0.9.3) in Python (version 3.9). The specific parameters are listed in Appendix Tables S6–S9. First, descriptive statistics were performed to examine the distribution situation of the sample. Second, we conducted the causal inference and influencing mechanism analyses through the Double Machine Learning (DML) based on random forest (RF). In our mediation analysis, we utilized the DML framework to estimate the causal pathways, specifically focusing on the mediation effect through identified mediators (28). Control variables and province fixed effect were added to further address potential confounders and omitted variable bias. Furthermore, a series of robustness tests were conducted to assess the stability of the impact of aging anxiety on healthcare utilization. A placebo test by randomly generating pseudo-treatment groups through 500 Monte Carlo permutations was also carried out. Third, heterogeneity analyses based on DML models were conducted to examine whether the relationship between aging anxiety and healthcare utilization is different across subgroups defined by other demographic characteristics or health status.

## 3 Results

### 3.1 Descriptive analysis

The descriptive statistics for the key variables in this study are summarized in Tables 1, 2. Table 1 presents the aging anxiety status across three dimensions. The mean score for aging anxiety was 2.374 (SD = 0.949), suggesting moderate levels of anxiety regarding aging-related concerns. This overall score is influenced by four key dimensions: health anxiety, which had a mean score of 2.633 (SD = 1.116), reflecting moderate concern about health issues; self-determination anxiety, with a mean of 2.265 (SD = 1.134), indicating concerns about personal autonomy; and financial anxiety, which scored a mean of 2.225 (SD = 1.201), showing moderate anxiety about financial stability (Table 1). Healthcare utilization was measured using a six-category ordinal scale, with 30.8% of respondents indicating no healthcare visits in the past year, and 35.7% reporting seeking care several times a year. For health-related perceptions, 52.2% of participants rated their sleep quality as relatively good, while 35.5% considered themselves to be physically healthy. In terms of psycho-logical well-being, 39.4% of respondents reported no feelings of depression, with 5.2% indicating severe physical deterioration. Significant levels of accessibility anxiety and affordability anxiety were observed, with substantial proportions of the population expressing concerns about healthcare access and the affordability of medical services. Self-efficacy loss in managing healthcare-related tasks was reported by 17.3% of individuals as being very unconfident (Table 2).

**Table 1:** The scores of different dimensions of aging anxiety ($\bar{X}$±S).

Dimension	Sample size	Minimum value	Maximum value	$\bar{X}$±S
Aging anxiety	2,663	1	5	2.374 ± 0.949
Health anxiety	2,663	1	5	2.633 ± 1.116
Self-determination anxiety	2,663	1	5	2.265 ± 1.134
Financial anxiety	2,663	1	5	2.225 ± 1.201

**Table 2:** Descriptive statistics.

Variable (percent)	Mean	SD	Median	IQR
Dependent variable				
Aging anxiety	2.370	0.949	2.333	1.333
Independent variable				
Healthcare utilization				
Never = 0(30.8%); once a year = 1(23.1%); several times a year = 2(35.7%); once a month = 3(7.7%); once a week = 4(1.8%); several times a week = 5(0.9%)	1.293	1.092	1	2
Mediating variables				
Sleep disorder				
Very good = 0(21.1%); relatively good = 1(52.2); relatively poor = 2(21.7); very poor = 3(5.0%)	1.105	0.786	1	1
Physical deterioration				
Very healthy = 0(17.6%); relatively healthy = 1(35.5%); average = 2(28.2%); relatively unhealthy = 3(13.5%); Very unhealthy = 4(5.2%)	1.532	1.087	1	1
Psychological pessimism				
Never = 0(39.4%); rarely = 1(26.7%); sometimes = 2(23.5%); often = 3(8.2%); always = 4(2.3%)	1.073	1.076	1	2
Accessibility anxiety				
Not worried at all = 0(14.2%); somewhat worried = 1(22.7%); Not very worried = 2(33.4%); very worried = 3(29.8%)	1.788	1.023	2	2
Affordability anxiety				
Not worried at all = 0(9.3%); somewhat worried = 1(15.8%); Not very worried = 2(29.9%); very worried = 3(44.9%)	2.104	0.985	2	2
Self-efficacy loss				
Very confident = 0(32.7%); confident = 1(15.1%); neither confident nor unconfident = 2(21.7%); Unconfident = 3(13.3%); Very unconfident = 4(17.3%)	1.673	1.475	2	3
Control variables				
Gender				
Male = 0(45.1%); female = 1(54.9%)	0.550	0.498	NA	NA
Age				
Youth (18–44) = 0(34.4%); middle-aged (45–60) = 1(28.9%); older adults (60 and above) = 2(36.8%)	1.020	0.843	1	2
Religion				
No religious belief = 0(91.3%); having religious beliefs = 1(8.7%)	0.090	0.282	NA	NA
Household				
Non-agricultural = 0(40.0%); Agricultural = 1(60.0%)	0.600	0.490	NA	NA
Marital				
Unmarried = 0(27.6%); Married = 1(72.4%)	0.720	0.447	NA	NA
Education				
Illiterate = 0(11.0%); literate = 1(89.0%)	0.586	0.806	NA	NA
Political				
Non-party member = 0(87.8%); Party member = 1(12.2%)	0.122	0.327	NA	NA
Kid number	1.690	1.262	2	1
Living environment	1.851	0.577	2.000	0.750

### 3.2 The influence of aging anxiety on healthcare utilization

By dividing the sample ratio at 1:4, Table 3 identifies the influence of aging anxiety on healthcare utilization through DML framework based on RF. In Mode 1 and Model 2, we only controlled province dummy variable or control variables. Both regression coefficients are remarkably positive at the 1% level. In Mode 3, we added both control variables and province dummy variable, finding that the coefficient is also positive at the 1% level (*β* = 0.110, *t* = 4.895). It robustly confirms that healthcare utilization would become more frequent with the enhancement of aging anxiety.

**Table 3:** The association between aging anxiety and healthcare utilization.

Variable	(1)	(2)	(3)
	Healthcare utilization	Healthcare utilization	Healthcare utilization
Aging anxiety	0.110***	0.111***	0.110***
	(4.877)	(4.909)	(4.895)
Control variables	N	Y	Y
Province FE	Y	N	Y
Obs.	2,663	2,663	2,663
DML	RF	RF	RF

1. *** *p* < 0.01, ** *p* < 0.05, * *p* < 0.1. 2. *t* statistics in parentheses. 3. The regressions include constant terms but are not reported.

To test the robustness of baseline regression, we conducted a series of analyses in Table 4. In Model 1, we substituted the previous measurement of aging anxiety with the summing scores of those three items (health anxiety, financial anxiety, and self-determination anxiety) related to aging anxiety. In Model 2, we added some control variables of social security, including basic endowment insurance, basic medical insurance, commercial endowment insurance, and commercial health insurance to prevent instability caused by omitted variables. In Model 3, we performed 5,000 bootstrap sampling tests to validate the reliability of statistical inferences. In Model 4, we removed municipality’s samples to avoid bias from their distinct economic, policy and resource gaps with other provinces. In Model 5, we changed K folds of cross-fitting to 1:9. In Model 6, the baseline method was replaced with DML based on Linear Regression (LR). Across all results, the coefficients constantly remain significant at the 1% level, thereby identifying the structural robustness of the enhancement effect of aging anxiety on healthcare utilization.

**Table 4:** The result of robustness test.

Variable	(1)	(2)	(3)	(4)	(5)	(6)
	Replace measurement	Add control variables	Bootstrap test	Remove municipality	Change K folds	Replace DML
Aging anxiety	0.037***	0.108***	0.111***	0.109***	0.112***	0.109***
	(4.947)	(4.692)	(4.914)	(4.554)	(4.985)	(4.836)
Control variables	Y	Y	Y	Y	Y	Y
Province FE	Y	Y	Y	Y	Y	Y
Obs.	2,663	2,538	2,663	2,199	2,663	2,663
DML	RF	RF	RF	RF	RF	LR

1. *** *p* < 0.01, ** *p* < 0.05, * *p* < 0.1. 2. *t* statistics in parentheses. 3. The regressions include constant terms but are not reported. 4. The dependent variables are healthcare utilization in Table 4.

**Figure 2:**
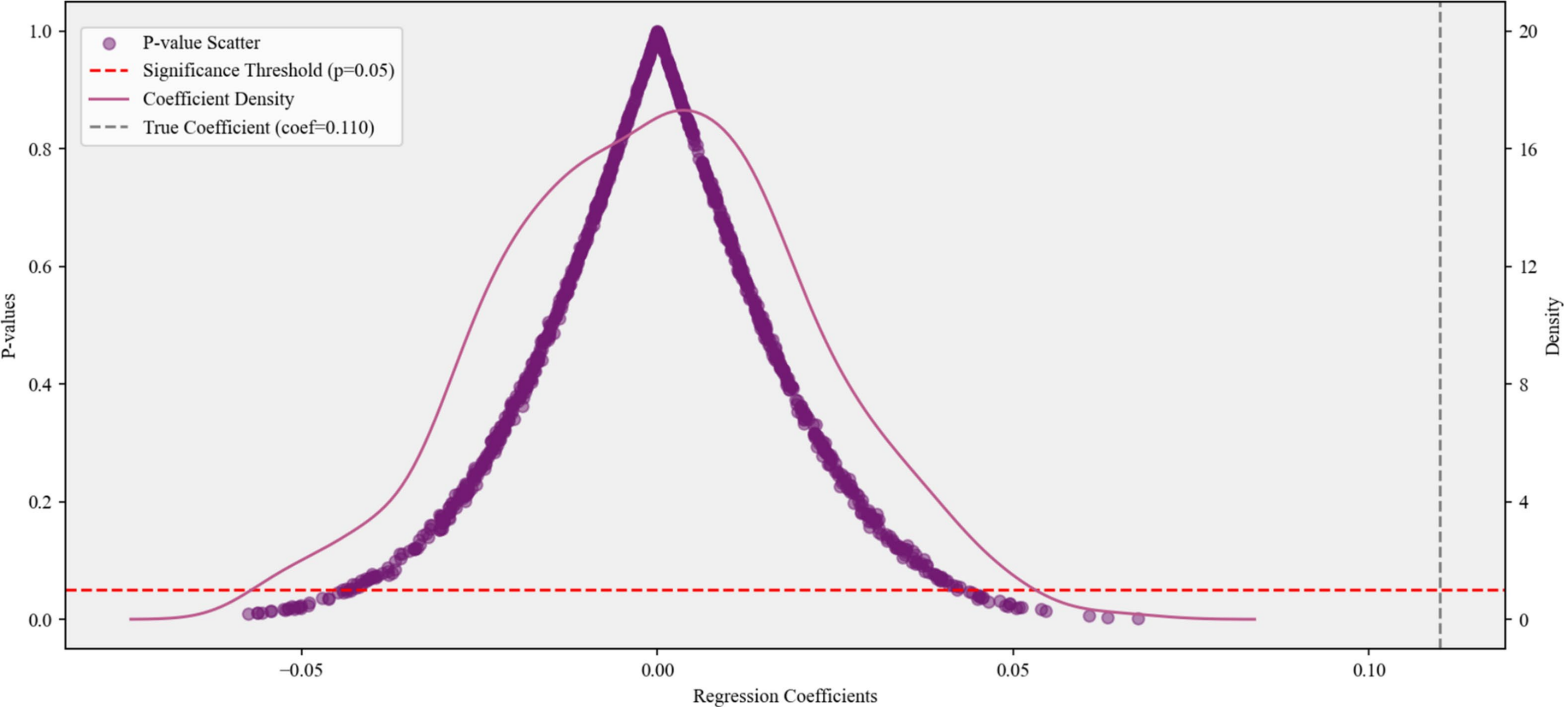
The result of placebo test.

Simultaneously, we also performed a placebo test by randomly generating pseudo-treatment groups through 1,000 Monte Carlo permutations. In Figure 2, the pseudo-treatment groups’ coefficients are predominantly centered around 0, with their estimated *p*-values mostly exceeding 0.05. A distinct separation between placebo effects and the actual coefficient value (0.110) is also obvious in kernel density estimation. We find no evidence of random-grouping bias, and the result of baseline regression is almost impossible to originate from random variations.

### 3.3 The influencing mechanism

In Table 5, we identify the two-dimensions influencing mechanisms of healthcare deprivation based on the theoretical framework. It verifies that healthcare deprivation is a significant pathway to induce healthcare utilization behavior of those individuals with aging anxiety.

**Table 5:** The influencing mechanisms of aging anxiety and healthcare utilization.

Variable	(1)	(2)	(3)	(4)	(5)	(6)
	Healthcare service deprivation		Healthcare condition deprivation			
	Accessibility anxiety	Affordability anxiety	Physical deterioration	Psychological pessimism	Sleep disorder	Self-efficacy loss
Aging anxiety	0.344***	0.384***	0.160***	0.175***	0.104***	0.160***
	(16.904)	(19.845)	(7.286)	(7.819)	(6.124)	(5.595)
Control variables	Y	Y	Y	Y	Y	Y
Province FE	Y	Y	Y	Y	Y	Y
Observations	2,663	2,663	2,663	2,663	2,663	2,663
DML	RF	RF	RF	RF	RF	RF

1. *** *p* < 0.01, ** *p* < 0.05, * *p* < 0.1. 2. *t* statistics in parentheses. 3. The regressions include constant terms but are not reported.

In Model 1 and Model 2, we found that each unit increase in aging anxiety can resulted in 0.344 increase in healthcare service accessibility anxiety and 0.384 increase in healthcare service affordability anxiety. This, called healthcare service deprivation, further heightens concerns over future access to essential health resources, triggering loss aversion-based preventative tendencies. In this condition, the fear of unmanaged health decline outweighs the costs of early intervention. Therefore, individuals amplify proactive healthcare utilization, seeking timely screening and care to address potential issues before they escalate into more severe problems.

In Model 3–6, the results unveiled that aging anxiety is an important cause of structural health deterioration, aggravating physical deterioration (*β* = 0.160, *t* = 7.286), psychological pessimism (*β* = 0.175, *t* = 7.819), sleep disorder (*β* = 0.104, *t* = 6.124), and self-efficacy loss (*β* = 0.160, *t* = 5.595). These interrelated outcomes, characterized by tangible health impairments and diminished psychological resilience, create urgent health signals that necessitate clinical intervention. It can be called healthcare condition deprivation. As physical discomfort intensifies, emotional distress persists, and functional capacities decline, individuals are compelled to seek diagnostic and therapeutic healthcare utilization to alleviate symptoms, address underlying health issues, and re-store daily functioning. This directly driving the increase in healthcare utilization for treatment purposes.

### 3.4 Heterogeneity analysis

**Figure 3:**
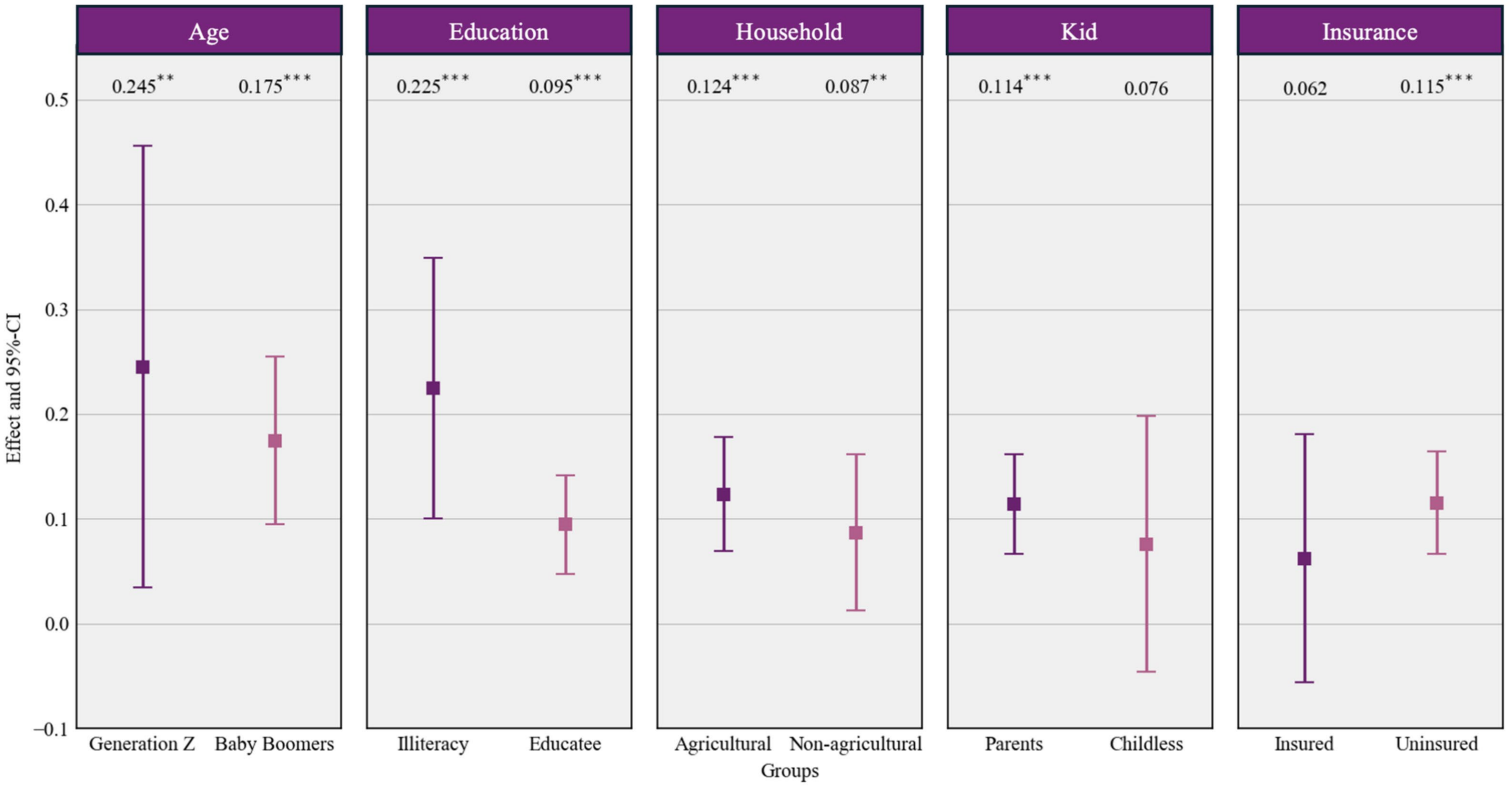
The result of heterogeneity test.

To assess variability in effects across population subgroups, we conducted a heterogeneity analysis. Figure 3 visually summarized the results through forest plots comparing effect sizes and 95% confidence intervals (CIs) across five socio-demographic categories.

Given the aging effect, generation Z exhibits a substantially stronger effect (*β* = 0.245, *p* < 0.01) compared to Baby Boomers (*β* = 0.175, *p* < 0.001), indicating greater susceptibility among younger cohorts. Simultaneously, consistent pattern emerges among vulnerable subgroups. Illiterate individuals (*β* = 0.225, *p* < 0.001) and agricultural house-holds (*β* = 0.124, *p* < 0.001) all show markedly elevated effects relative to their more advantaged counterparts. This demonstrates the protective role of education and socio-economic privilege in buffering against aging anxiety-driven healthcare utilization. Notably, intergenerational support further amplifies utilization responses, with parents exhibiting significant anxiety-driven healthcare utilization (*β* = 0.114, *p* < 0.001) compared to childless individuals who showed no statistically significant effect. Interestingly, aging anxiety significantly increases healthcare utilization among the uninsured (*β* = 0.115, *p* < 0.001), while no significant effect emerged among the insured. This underscores that lack of insurance coverage heightens vulnerability, transforming aging anxiety directly into healthcare seeking behavior, contrasting with the buffering effect of having insurance.

## 4 Discussion

### 4.1 Summary of findings

The intensification of an individual’s aging anxiety is significantly associated with an increase in their healthcare utilization. Concerns about financial instability caused by aging, dependence on others for care, or physical disability constitute a persistent psychological stressor (29). This stressor may through mind–body interactions heighten an individual’s sensitivity to current physical discomforts and may genuinely increase certain stress-related psychosomatic health risks (30), all of which can lead to more frequent seeking of medical services. Furthermore, perceived future risks may strongly activate an individual’s preventive motivation (31). Out of a desire to avoid premature health decline, individuals with higher aging anxiety are more likely to actively use medical services in an attempt to gain a sense of control and reduce future health and financial burdens through early intervention. At the same time, anxiety surrounding economic security and loss of autonomy may also drive individuals to maintain optimal health in order to delay the onset of dependence, thereby increasing their demand for various medical services. Therefore, multidimensional anxiety about aging is not merely reflected at the emotional level; it may also influence individuals’ medical decision-making behaviors (32) and serve as a key factor driving increased utilization of healthcare services.

The research findings also indicate that the promotive effect of aging anxiety on healthcare utilization is partly realized through the amplification of healthcare deprivation. The Theory of Care-Seeking Behavior posits that individuals’ decisions to seek medical care result from an evaluation of symptom severity, attribution, perceived threat of illness, and a cost–benefit analysis of taking action (33). Aging anxiety not only directly prompts individuals to seek medical care, but also influences their perception and assessment of their medical circumstances and health status. Healthcare deprivation encompasses concerns over the accessibility and financial burden of healthcare services, as well as negative perceptions of one’s own health condition and a sense of lost capability. Aging anxiety magnifies deprivation in both these dimensions, thereby driving care-seeking behavior. Previous studies in developed countries such as the United States have highlighted that aging anxiety leads to increased healthcare utilization, particularly as individuals become more emotionally distressed about aging (34). However, our study offers a new perspective by suggesting that aging anxiety in China is not only driven by emotional distress but also by significant healthcare deprivation, where limited access to medical services exacerbates the anxiety, prompting individuals to seek care more proactively. This distinguishes our findings from those in developed countries, where healthcare access is generally more reliable.

When individuals experience strong aging anxiety, they tend to amplify vigilance toward health risks and fears regarding insufficient resources. This anxious state in-creases sensitivity to subtle bodily changes or discomforts. Aging anxiety may elicit existential concerns (35), making individuals more likely to interpret such signals as signs of serious health problems. Based on loss aversion tendencies (36), individuals prioritize avoiding significant future losses caused by health deterioration, relatively downplaying the costs and efforts required to seek current medical attention. Consequently, concerns about potentially losing control over health and future basic medical resource security translate into a proactive motivation to avoid risks (37). This drives individuals to actively seek medical interventions, manifested in increased use of both preventive and curative services. At the same time, anxiety undermines individuals’ confidence in self-managing their health, making problems feel beyond their coping capacity. On the other hand, aging anxiety exacerbates worries about potential barriers to accessing medical resources, leading individuals to anticipate and amplify future medical needs, payment ability, and service accessibility issues. Andersen’s Behavioral Model of Health Services Use (38) indicates that individuals’ decisions to seek services are driven by an integration of enabling and need factors. The combined effect of these two heightened senses of deprivation forms a psychological state in which health is perceived as genuinely threatened and coping resources are potentially insufficient. It is precisely this intensified psychological state, integrating concerns about health status and resource accessibility, that constitutes the intrinsic motivation driving individuals to seek healthcare services.

Heterogeneity analysis examined the variability of aging anxiety’s impact on healthcare utilization across different population subgroups and analyzed the moderating effects of sociodemographic characteristics.

Generation Z exhibits a significantly stronger response to aging anxiety compared to the Baby Boomer generation, which aligns with previous research findings (6). A possible explanation is that younger cohorts face longer-term uncertainties, including concerns about the sustainability of future pension systems, a lack of long-term confidence in their ability to accumulate healthcare resources, and fear of potentially high treatment costs resulting from delayed health investments. This heightened sensitivity to “long-term risks” causes anxiety to more directly translate into current demand for preventive and curative healthcare services (39). Moreover, China’s educational system and that of many Western countries significantly differ, particularly in terms of access and equity. In China, despite the growing availability of higher education, disparities in access to quality education remain, especially between urban and rural areas (40). This educational gap can affect individuals’ understanding of healthcare and aging issues, shaping their perception of aging anxiety. In contrast, cohort studies in Portugal and Northern Europe demonstrate that systematic health literacy interventions can significantly enhance well-being among older adults while reducing anxiety/depression symptoms (41, 42). Many Western countries have more robust public education systems and healthcare literacy programs that help alleviate some of the psychological burdens linked to aging anxiety.

A striking difference in our findings is that, while younger generations in developed countries express similar concerns about long-term health risks, our study reveals that in China, these concerns are heightened due to uncertainties surrounding the sustainability of the healthcare system and the lack of social security mechanisms. As a result, the reaction to aging anxiety is stronger in China, particularly among younger cohorts who face greater insecurity about future healthcare resources. This adds another layer of complexity to how aging anxiety is experienced, with the combination of educational disparities and healthcare system uncertainties amplifying the response in younger generations.

Among low socioeconomic status groups (including illiterate populations and rural households), aging-anxiety-driven healthcare utilization is significantly higher than in higher socioeconomic status groups. Deficiencies in education and financial resources weaken individuals’ capacity and confidence to cope with health risks. Low educational attainment may limit access to health information, risk assessment ability, and effective resource mobilization (43); meanwhile, economic scarcity amplifies fears regarding the burden of medical expenses (44). Faced with comparable levels of aging anxiety, low socioeconomic status individuals, lacking buffers such as savings or diversified support networks (45), are more likely to convert anxiety into urgent behaviors to seek immediate medical resources to mitigate present uncertainties and future risks.

The COVID-19 pandemic has further exacerbated these inequalities, as individuals from lower socioeconomic backgrounds often face greater difficulties in accessing both healthcare and educational resources (46). In many developing countries, including China, the pandemic disrupted education and limited the dissemination of critical health information, which disproportionately affected marginalized groups (47). This disruption may have further amplified aging anxiety, as individuals feel more uncertain about their healthcare options and the future availability of medical services. Beyond the impact of COVID-19, other unincorporated variables such as regional healthcare infrastructure, access to digital health tools, and variations in social support systems may further influence healthcare-seeking behaviors (48). For instance, individuals in rural or underserved areas might experience heightened anxiety due to limited access to healthcare resources, while changes in mental health awareness could also affect individuals’ responses to aging anxiety (49). These additional factors warrant further exploration to offer a more holistic understanding of the drivers behind aging-related healthcare utilization.

Compared with childless individuals, parents’ anxiety significantly enhances their healthcare utilization behaviors, reflecting the motivational mechanism of family responsibility (50). Parents are more inclined to proactively intervene on their aging-related health concerns, driven by the desire to fulfill ongoing caregiving duties and avoid becoming a burden to their children. In contrast, childless individuals may experience relatively lower levels of anxiety-driven healthcare utilization due to the absence of such direct responsibility pressures or differing dependency pathways on socialized eldercare services.

Medical insurance plays a protective role. The health conditions arising during aging are closely related to social security policies (51). Lack of insurance means individuals are directly exposed to potential catastrophic medical financial risks (52), which may heighten their sensitivity and fear of aging-related health decline. Anxiety is often interpreted as the pressure of potentially high medical expenses, thereby strongly driving care-seeking behavior. Conversely, insurance coverage provides financial buffering and psychological security, reducing the motivation to seek premature or excessive medical services out of fear of costs (53).

### 4.2 Policy implications

Based on these findings, policy interventions need to build a multidimensional response system. First, strengthen the safety net function of medical insurance by expanding outpatient coverage and improving convenience in cross-regional settlement. Develop transitional enrollment incentive policies for young migrants and informal employees to alleviate their economic burden anxiety by reducing their actual out-of-pocket costs. Second, add older adults health management service packages in primary healthcare institutions, integrating basic physical examinations, chronic disease screening, and psychological assessments to replace passive healthcare-seeking with proactive services. Third, implement health literacy enhancement initiatives by providing low-education groups with healthcare navigation, insurance configuration guidance, and self-health management training through community health stations, reducing service accessibility anxiety caused by information asymmetry. Last but not least, explore models of joint family and community support by encouraging children’s participation in parental health management and constructing community mutual aid eldercare networks for the childless, filling the motivation gap in preventive healthcare behavior caused by family absence. In summary, dissipating the material basis of deprivation through institutional design facilitates the alleviation of medical re-source-related anxiety and the synergistic realization of healthy aging goals.

### 4.3 Limitations

Admittedly, this study has certain limitations. Restricted by the CGSS database content, there remains room to deepen the measurement of core dimensions of variables such as aging anxiety and Healthcare Deprivation. Additionally, relying on cross-sectional data to identify mechanisms suggests that future research could com-bine longitudinal surveys or experimental studies to further validate the dynamic evolution and causal temporality of the mediating pathways.

## 5 Conclusion

The study reveals the complex psychosocial mechanisms underlying healthcare demand and utilization in the aging process. Using large-scale CGSS data, the research systematically examined the impact and mechanisms of aging anxiety on individual healthcare utilization through a double machine learning approach. Aging anxiety is not only a health concern but also a stress response to the structural risk of insufficient medical resources. The heightened sensitivity observed in younger cohorts mirrors a crisis of trust in the long-term health security system.

## Data Availability

The original contributions presented in the study are included in the article/Supplementary material, further inquiries can be directed to the corresponding author.

## References

[1] Department of Economic. World Population Prospects 2024: Summary of Results. Stylus Publishing, LLC. (2024).

[2] Lasher KP Faulkender PJ. Measurement of aging anxiety: development of the anxiety about aging scale. Int J Aging Hum Dev. (1993) 37:247–59. doi: 10.2190/1U69-9AU2-V6LH-9Y1L, PMID: 8307644

[3] McConatha JT Schnell F Volkwein K Riley L Leach E. Attitudes toward aging: a comparative analysis of young adults from the United States and Germany. Int J Aging Hum Dev. (2003) 57:203–15. doi: 10.2190/K8Q8-5549-0Y4K-UGG0, PMID: 15176668

[4] Levy B. Stereotype embodiment: a psychosocial approach to aging. Curr Dir Psychol Sci. (2009) 18:332–6. doi: 10.1111/j.1467-8721.2009.01662.x 20802838 PMC2927354

[5] Wang D Chonody J. Social workers’ attitudes toward older adults: a review of the literature. J Soc Work Educ. (2013) 49:150–72. doi: 10.1080/10437797.2013.755104

[6] Lynch SM. Measurement and prediction of aging anxiety. Res Aging. (2000) 22:533–58. doi: 10.1177/0164027500225004

[7] Barnett MD Adams CM. Ageism and aging anxiety among young adults: relationships with contact, knowledge, fear of death, and optimism. Educ Gerontol. (2018) 44:693–700. doi: 10.1080/03601277.2018.1537163

[8] Kruger A. The midlife transition—crisis or chimera. Psychol Rep. (1994) 75:1299–305. doi: 10.2466/pr0.1994.75.3.1299, PMID: 7892395

[9] Fergus TA Griggs JO Cunningham SC Kelly LP. Health anxiety and medical utilization: the moderating effect of age among patients in primary care. J Anxiety Disord. (2017) 51:79–85. doi: 10.1016/j.janxdis.2017.06.002 28689676

[10] Diehr P Yanez D Ash A Hornbrook M Lin DY. Methods for analyzing health care utilization and costs. Annu Rev Public Health. (1999) 20:125–44. doi: 10.1146/annurev.publhealth.20.1.125, PMID: 10352853

[11] Yawar R Khan S Rafiq M Fawad N Shams S Navid S. Aging is inevitable: understanding aging anxiety related to physical symptomology and quality of life with the mediating role of self-esteem in adults. Int J Hum Rights Healthc. (2024) 17:170–85. doi: 10.1108/IJHRH-05-2022-0047

[12] Martínez-Jiménez M Hollingsworth B Zucchelli E. Socioeconomic deprivation, health and healthcare utilisation among millennials. Soc Sci Med. (2024) 351:116961. doi: 10.1016/j.socscimed.2024.116961, PMID: 38761457

[13] Musich S Wang S Hawkins K Klemes A. The impact of personalized preventive care on health care quality, utilization, and expenditures. Popul Health Manag. (2016) 19:389–97. doi: 10.1089/pop.2015.0171, PMID: 26871762 PMC5296930

[14] Remmers C Hibbard J Mosen DM Wagenfield M Hoye RE Jones C. Is patient activation associated with future health outcomes and healthcare utilization among patients with diabetes? J Ambul Care Manage. (2009) 32:320–7. doi: 10.1097/JAC.0b013e3181ba6e77 19888008

[15] Gilthorpe MS Wilson RC. Rural/urban differences in the association between deprivation and healthcare utilisation. Soc Sci Med. (2003) 57:2055–63. doi: 10.1016/S0277-9536(03)00071-6 14512237

[16] Kruk ME Gage AD Arsenault C Jordan K Leslie HH Roder-DeWan S. High-quality health systems in the sustainable development goals era: time for a revolution. Lancet Glob Health. (2018) 6:e1196–252. 30196093 10.1016/S2214-109X(18)30386-3 PMC7734391

[17] Yun R. J. Lachman M. E. (2006). Perceptions of aging in two cultures: Korean and American views on old age. J Cross Cult Gerontol, 21: 55–70. 17103311 10.1007/s10823-006-9018-y

[18] Subramaniam M Sum CF Pek E Stahl D Verma S Liow PH. Comorbid depression and increased health care utilisation in individuals with diabetes. Gen Hosp Psychiatry. (2009) 31:220–4. doi: 10.1016/j.genhosppsych.2009.01.001, PMID: 19410100

[19] Becker TD. Health care utilization as a predictor of death anxiety in older adults. Death Stud. (2022) 46:728–37. doi: 10.1080/07481187.2020.1774942, PMID: 32615057

[20] Gu H Tan Q Guo Y He H Zhang Y. Family support, social security, commercial insurance, and aging anxiety among Chinese residents: a study based on the 2021 CGSS data. Front Public Health. (2025) 13:1577384. doi: 10.3389/fpubh.2025.1577384, PMID: 40265070 PMC12013337

[21] Knaus MC. Double machine learning-based programme evaluation under unconfoundedness. Econom J. (2022) 25:602–27. doi: 10.1093/ectj/utac015

[22] Chernozhukov V Chetverikov D Demirer M Duflo E Hansen C Newey W. Double/debiased machine learning for treatment and structural parameters. Econom J. (2018) 21:C1–C68. doi: 10.1111/ectj.12097

[23] Han J Zhao X. Impact of internet use on multi-dimensional health: an empirical study based on CGSS 2017 data. Front Public Health. (2021) 9:749816. doi: 10.3389/fpubh.2021.749816, PMID: 34733815 PMC8558342

[24] Zhu F Tan B Jang Y. Internet use and self-rated health: the mediating role of physical exercise. Healthcare (Basel). (2025) 13:714. doi: 10.3390/healthcare13070714, PMID: 40218012 PMC11988760

[25] Pifer MA Segal DL. On the measurement of aging anxiety: comparative validity of two popular measures among older adults. Int J Aging Hum Dev. (2025) 101:171–88. doi: 10.1177/00914150241260828, PMID: 38859731

[26] Laranjeira C Querido A. Hope and optimism as an opportunity to improve the “positive mental health” demand. Front Psychol. (2022) 13:827320. doi: 10.3389/fpsyg.2022.827320, PMID: 35282230 PMC8907849

[27] Abolfathi Momtaz Y Ibrahim R Hamid TA. The impact of giving support to others on older adults’ perceived health status. Psychogeriatrics. (2014) 14:31–7. doi: 10.1111/psyg.12036, PMID: 24299124

[28] Farbmacher H Huber M Lafférs L Langen H Spindler M. Causal mediation analysis with double machine learning. Econom J. (2022) 25:277–300. doi: 10.1093/ectj/utac003

[29] Cohen BE Edmondson D Kronish IM. State of the art review: depression, stress, anxiety, and cardiovascular disease. Am J Hypertens. (2015) 28:1295–302. doi: 10.1093/ajh/hpv047, PMID: 25911639 PMC4612342

[30] Abdelall ES Eagle Z Finseth T Mumani AA Wang Z Dorneich MC. The interaction between physical and psychosocial stressors. Front Behav Neurosci. (2020) 14:63. doi: 10.3389/fnbeh.2020.00063, PMID: 32528259 PMC7247805

[31] Chapman GB Brewer NT Coups EJ Brownlee S Leventhal H Levanthal EA. Value for the future and preventive health behavior. J Exp Psychol Appl. (2001) 7:235–50. doi: 10.1037/1076-898X.7.3.235 11676102

[32] Poortaghi S Raiesifar A Bozorgzad P Golzari SEJ Parvizy S Rafii F. Evolutionary concept analysis of health seeking behavior in nursing: a systematic review. BMC Health Serv Res. (2015) 15:523. doi: 10.1186/s12913-015-1181-9, PMID: 26613729 PMC4662038

[33] Lauver DA. Theory of care-seeking behavior. Int J Nurs Stud. (1992) 24:281–7. doi: 10.1111/j.1547-5069.1992.tb00734.x 1452182

[34] Brenes GA Penninx BW Judd PH Rockwell E Sewell DD Wetherell JL. Anxiety, depression and disability across the lifespan. Aging Ment Health. (2008) 12:158–63. doi: 10.1080/13607860601124115, PMID: 18297491 PMC2879640

[35] Bergman YS Bodner E. Aging anxiety in older adults. GeroPsych. (2022) 35:196–201. doi: 10.1024/1662-9647/a000295

[36] Loewenstein GF Weber EU Hsee CK Welch N. Risk as feelings. Psychol Bull. (2001) 127:267–86. doi: 10.1037/0033-2909.127.2.267 11316014

[37] Razavi SA Shahrabi A Siamian H. The relationship between research anxiety and self-efficacy. Mater Sociomed. (2017) 29:247–50. doi: 10.5455/msm.2017.29.247-250, PMID: 29284993 PMC5723194

[38] Andersen RM. National health surveys and the behavioral model of health services use. Med Care. (2008) 46:647–53. doi: 10.1097/MLR.0b013e31817a835d 18580382

[39] Prins MA Verhaak PF Bensing JM van der Meer K. Health beliefs and perceived need for mental health care of anxiety and depression--the patients’ perspective explored. Clin Psychol Rev. (2008) 28:1038–58. doi: 10.1016/j.cpr.2008.02.009 18420323

[40] Zheng X Xu Z Zhao J Hao S Xu F Ding S. Disparities in anxiety and related factors among Chinese older adults across different aged-care models: a comparison of two cross-sectional studies. BMC Geriatr. (2025) 25:46. doi: 10.1186/s12877-024-05653-3, PMID: 39838310 PMC11748326

[41] Sadio R Henriques A Nogueira P Costa A. A multidimensional analysis of older adults wellbeing and health literacy in Alentejo: a cross-sectional study. Front Public Health. (2025) 13:1514968. doi: 10.3389/fpubh.2025.1514968, PMID: 40352857 PMC12061670

[42] Gustafsdottir SS Sigurdardottir AK Mårtensson L Arnadottir SA. Making Europe health literate: including older adults in sparsely populated Arctic areas. BMC Public Health. (2022) 22:511. doi: 10.1186/s12889-022-12935-1, PMID: 35296283 PMC8924562

[43] Richardson A Allen JA Xiao H Vallone D. Effects of race/ethnicity and socioeconomic status on health information-seeking, confidence, and trust. J Health Care Poor Underserved. (2012) 23:1477–93. doi: 10.1353/hpu.2012.0181, PMID: 23698662

[44] Epstein AM Stern RS Weissman JS. Do the poor cost more? A multihospital study of patients’ socioeconomic status and use of hospital resources. N Engl J Med. (1990) 322:1122–8. doi: 10.1056/NEJM199004193221606 2108331

[45] Schafer MH Vargas N. The dynamics of social support inequality: maintenance gaps by socioeconomic status and race? Soc Forces. (2016) 94:1795–822. doi: 10.1093/sf/sow024

[46] Frey A Tilstra AM Verhagen MD. Inequalities in healthcare use during the COVID-19 pandemic. Nat Commun. (2024) 15:1894. doi: 10.1038/s41467-024-45720-2, PMID: 38424038 PMC10904793

[47] World Health Organization. Operational framework for monitoring social determinants of health equity. Geneva: World Health Organization (2024).

[48] Yip W Ge L Ho AHY Heng BH Tan WS. Building community resilience beyond COVID-19: the Singapore way. Lancet Reg Health West Pac. (2021) 7:100091. doi: 10.1016/j.lanwpc.2020.100091, PMID: 33521745 PMC7825823

[49] Debsarma D Choudhary BK. Exploring the socio-ecological factors of healthcare-seeking behaviour among patients/people from rural unqualified health providers in the rural settings in West Bengal, India. SSM Health Syst. (2025) 4:100046. doi: 10.1016/j.ssmhs.2024.100046

[50] Apetroaia A Hill C Creswell C. Parental responsibility beliefs: associations with parental anxiety and behaviours in the context of childhood anxiety disorders. J Affect Disord. (2015) 188:127–33. doi: 10.1016/j.jad.2015.08.059, PMID: 26363612 PMC4641868

[51] De Oliveira Teixeira LM Uribe FAR Moreira HLF da Silva Pedroso J. Associations between retirement, social security policies and the health of older people: a systematic review. BMC Public Health. (2024) 24:2473. doi: 10.1186/s12889-024-19979-5 39261849 PMC11389105

[52] Dou G Wang Q Ying X. Reducing the medical economic burden of health insurance in China: achievements and challenges. Biosci Trends. (2018) 12:215–9. doi: 10.5582/bst.2018.01054, PMID: 29925702

[53] Shu Z Han Y Xiao J Li J. Effect of medical insurance and family financial risk on healthcare utilisation by patients with chronic diseases in China: a cross-sectional study. BMJ Open. (2019) 9:e030799. doi: 10.1136/bmjopen-2019-030799, PMID: 31748294 PMC6887032

