# Supplementary material for: Healthcare deprivation matters: a novel framework to unveil the influencing mechanisms of aging anxiety and healthcare utilization
Source: Front Public Health. 2025 Oct 14;13:1679262. doi: 10.3389/fpubh.2025.1679262 (PMC12558805; doi:10.3389/fpubh.2025.1679262)
Supplement: Supplementary file 1 [file Supplementary_file_1.docx]

# Appendix

Table S1. Variable measurement.

| **Variable** | **Measurement** |
| --- | --- |
| ***Dependent Variable*** | |
| Healthcare utilization | “How often did you seek medical care for your own health in the past year?”  Never = 0; Once a year = 1; Several times a year = 2; Once a month = 3; Once a week = 4; Several times a week = 5 |
| ***Independent Variable*** |  |
| Aging anxiety | This variable is a continuous variable by calculating the mean score of the responses to the following statements.   1. “I worry that I will not be able to take care of myself when I get old.” 2. “I worry that I will have to let others make decisions for me when I get old.” 3. “Being financially dependent on others is one of my biggest concerns about growing old.”   For each statement, respondents are asked, “To what extent do you agree with the statement?” The responses and their corresponding scores are: Strongly disagree = 0; Disagree = 1; Neither agree nor disagree = 2; Agree = 3; Strongly agree = 4. |
| ***Mediating Variables*** |  |
| Accessibility Anxiety | “Are you worried about being unable to access medical services when you need them?”  Not worried at all = 0; Somewhat worried = 1; Not very worried = 2; Very worried = 3 |
| Affordability Anxiety | “Are you worried about being unable to afford medical expenses when suffering from a serious illness?”  Not worried at all = 0; Somewhat worried = 1; Not very worried = 2; Very worried = 3 |
| Physical deterioration | “How would you describe your current physical health status?”  Very healthy = 0; Relatively healthy = 1; Average = 2; Relatively unhealthy = 3; Very unhealthy = 4 |
| Psychological pessimism | “How often do you feel depressed or down?”  Never = 0; Rarely = 1; Sometimes = 2; Often = 3; Always= 4 |
| Sleep disorder | “How would you rate your sleep quality?”  Very good = 0; Relatively good = 1; Relatively poor = 2; Very poor = 3 |
| Self-efficacy loss | “How confident are you in filling out medical forms such as personal profiles, medical histories, and informed consent forms?”  Very confident = 0; Confident = 1; Neither confident nor unconfident = 2; Unconfident = 3; Very unconfident = 4 |
| ***Control Variables*** |  |
| Age | Youth (18–44) = 0; Middle-aged (45–60) = 1; Elderly (60 and above) = 2 |
| Gender | Male = 0; Female = 1 |
| Religion | No religious belief = 0; Having religious beliefs=1 |
| Educational status | Less than lower secondary = 0; upper secondary & vocational training = 1; Tertiary = 2 |
| Marital status | Unmarried = 0; Married = 1 |
| Political status | Non-Party member = 0; Party member = 1 |
| Household | Non-agricultural = 0; Agricultural = 1 |
| Kid number | The sum of biological children, adopted children, and stepchildren reported by the respondent, with values as non-negative integers. |
| Living environment | This variable is a continuous variable by calculating the mean score of the responses to the following statements.   1. “What is the severity of air pollution in your place of residence?” 2. “What is the severity of water pollution in your place of residence?” 3. “What is the severity of noise pollution in your place of residence?” 4. “What is the severity of insufficient lighting in your place of residence?”   For each question, the response options and their corresponding scores are: Not severe at all = 0; Somewhat severe = 1; Severe = 2; Very severe = 3. |

Table S2. Pearson correlation test.

|  |  | Aging anxiety | Financial | Self-determination | Health |
| --- | --- | --- | --- | --- | --- |
| Aging anxiety | Pearson | 1 | .815** | .838** | .822** |
|  | P |  | 0 | 0 | 0 |
|  | N | 2663 | 2663 | 2663 | 2663 |
| Financial anxiety | Pearson | .815** | 1 | .550** | .482** |
|  | P | 0 |  | 0 | 0 |
|  | N | 2663 | 2663 | 2663 | 2663 |
| Self-determination anxiety | Pearson | .838** | .550** | 1 | .530** |
|  | P | 0 | 0 |  | 0 |
|  | N | 2663 | 2663 | 2663 | 2663 |
| Health anxiety | Pearson | .822** | .482** | .530** | 1 |
|  | P | 0 | 0 | 0 |  |
|  | N | 2663 | 2663 | 2663 | 2663 |
| ** At the 0.01 level (double tailed), the correlation is significant. | | | | | |

Table S3. KMO and Bartlett inspection.

| KMO sampling suitability quantity |  | 0.693 |
| --- | --- | --- |
| Bartlett sphericity test | Approximate chi-square | 2034.836 |
|  | df | 3 |
|  | Sig. | 0 |

Table S4. Explanation of Total Variance.

| Component | Initial eigenvalue | | | Extract the sum of squared loads | | |
| --- | --- | --- | --- | --- | --- | --- |
|  | Total | Variance percentage | Accumulated% | Total | Variance percentage | Accumulated% |
| 1 | 2.042 | 68.058 | 68.058 | 2.042 | 68.058 | 68.058 |
| 2 | 0.52 | 17.319 | 85.378 |  |  |  |
| 3 | 0.439 | 14.622 | 100 |  |  |  |
| Extraction method: Principal Component Analysis. | | | | | | |

Table S5. Component Matrixa.

|  | Component |
| --- | --- |
|  | 1 |
| Health anxiety | 0.821 |
| Self-determination anxiety | 0.845 |
| Financial anxiety | 0.809 |
| Extraction method: Principal Component Analysis. | |
| Extracted 1 component |  |

Table S6. Model Construction Parameters in Models 1-3 of Table 3, Models 1, 2, and 4 of Table 4, Models 1-6 of Table 5, placebo test in Figure 2 and Models 1-10 of Figure 3.

| Parameters | Value | Type |
| --- | --- | --- |
| *doubleml.plm.DoubleMLPLR* |  |  |
| ml_l | RandomForestRegressor() | sklearn.ensemble._forest.RandomForestRegressor |
| ml_m | RandomForestRegressor() | sklearn.ensemble._forest.RandomForestRegressor |
| ml_g | None | NoneType |
| n_folds | 5 | int |
| n_rep | 1 | int |
| score | partialling out | str |
| draw_sample_splitting | True | bool |
| *sklearn.ensemble._forest.RandomForestRegressor* |  |  |
| n_estimators | 700 | int |
| criterion | squared_error | str |
| max_depth | 3 | int |
| min_samples_split | 2 | int |
| min_samples_leaf | 1 | int |
| min_weight_fraction_leaf | 0.0 | float |
| max_features | log2 | str |
| max_leaf_nodes | None | NoneType |
| min_impurity_decrease | 0.0 | float |
| bootstrap | True | bool |
| oob_score | False | bool |
| n_jobs | None | NoneType |
| random_state | 42 | int |
| verbose | 0 | int |
| warm_start | False | bool |
| class_weight | None | NoneType |
| ccp_alpha | 0.0 | float |
| max_samples | None | NoneType |
| monotonic_cst | None | NoneType |

Table S7. Model Construction Parameters in Models 3 of Table 4.

| Parameters | Value | Type |
| --- | --- | --- |
| *doubleml.plm.DoubleMLPLR* |  |  |
| ml_l | RandomForestRegressor() | sklearn.ensemble._forest.RandomForestRegressor |
| ml_m | RandomForestRegressor() | sklearn.ensemble._forest.RandomForestRegressor |
| ml_g | None | NoneType |
| n_folds | 5 | int |
| n_rep | 1 | int |
| score | partialling out | str |
| draw_sample_splitting | True | bool |
| *doubleml.plm.DoubleMLPLR.bootstrap* |  |  |
| method | normal | str |
| n_rep_boot | 500 | int |
| *sklearn.ensemble._forest.RandomForestRegressor* |  |  |
| n_estimators | 700 | int |
| criterion | squared_error | str |
| max_depth | 3 | int |
| min_samples_split | 2 | int |
| min_samples_leaf | 1 | int |
| min_weight_fraction_leaf | 0.0 | float |
| max_features | log2 | str |
| max_leaf_nodes | None | NoneType |
| min_impurity_decrease | 0.0 | float |
| bootstrap | True | bool |
| oob_score | False | bool |
| n_jobs | None | NoneType |
| random_state | 42 | int |
| verbose | 0 | int |
| warm_start | False | bool |
| class_weight | None | NoneType |
| ccp_alpha | 0.0 | float |
| max_samples | None | NoneType |
| monotonic_cst | None | NoneType |

Table S8. Model Construction Parameters in Models 5 of Table 4.

| Parameters | Value | Type |
| --- | --- | --- |
| *doubleml.plm.DoubleMLPLR* |  |  |
| ml_l | RandomForestRegressor() | sklearn.ensemble._forest.RandomForestRegressor |
| ml_m | RandomForestRegressor() | sklearn.ensemble._forest.RandomForestRegressor |
| ml_g | None | NoneType |
| n_folds | 10 | int |
| n_rep | 1 | int |
| score | partialling out | str |
| draw_sample_splitting | True | bool |
| *sklearn.ensemble._forest.RandomForestRegressor* |  |  |
| n_estimators | 700 | int |
| criterion | squared_error | str |
| max_depth | 3 | int |
| min_samples_split | 2 | int |
| min_samples_leaf | 1 | int |
| min_weight_fraction_leaf | 0.0 | float |
| max_features | log2 | str |
| max_leaf_nodes | None | NoneType |
| min_impurity_decrease | 0.0 | float |
| bootstrap | True | bool |
| oob_score | False | bool |
| n_jobs | None | NoneType |
| random_state | 42 | int |
| verbose | 0 | int |
| warm_start | False | bool |
| class_weight | None | NoneType |
| ccp_alpha | 0.0 | float |
| max_samples | None | NoneType |
| monotonic_cst | None | NoneType |

Table S9. Model Construction Parameters in Models 5 of Table 4.

| Parameters | Value | Type |
| --- | --- | --- |
| *doubleml.plm.DoubleMLPLR* |  |  |
| ml_l | LinearRegression() | sklearn.linear_model._base.LinearRegression |
| ml_m | LinearRegression() | sklearn.linear_model._base.LinearRegression |
| ml_g | None | NoneType |
| n_folds | 5 | int |
| n_rep | 1 | int |
| score | partialling out | str |
| draw_sample_splitting | True | bool |
| *sklearn.linear_model._base.LinearRegression* |  |  |
| fit_intercept | True | bool |
| copy_X | True | bool |
| n_jobs | None | NoneType |
| positive | False | bool |
